# Assessment of pancreatitis associated with tocilizumab use using the United States Food and Drug Administration Adverse Event Reporting System database

**DOI:** 10.1038/s41598-021-98325-w

**Published:** 2021-09-22

**Authors:** Ashwin Kamath, Sahana D. Acharya, Rashmi R. Rao, Sheetal D. Ullal

**Affiliations:** Department of Pharmacology, Kasturba Medical College, Mangalore, Manipal Academy of Higher Education, Manipal, India

**Keywords:** Data mining, Pancreatic disease, Adverse effects, Drug therapy, Rheumatic diseases

## Abstract

Tocilizumab (TCZ) is used to treat rheumatoid arthritis and other systemic inflammatory disorders. There is some evidence suggesting the occurrence of pancreatitis following TCZ use. We aimed to determine the reporting of pancreatitis following TCZ use in comparison with other drugs using the United States Food and Drug Administration Adverse Event Reporting System (FAERS) database. We extracted adverse event reports submitted to FAERS during 2013–2019. A reporting odds ratio (ROR) with the lower bound 95% confidence interval (CI) > 1 and a lower limit of a two-sided 95% interval of information component (IC_025_) more than zero was considered significant. Following deduplication, 3,383,910 adverse event reports were available; 144 (0.004%) reports were of pancreatic adverse events associated with TCZ use, and 15,907 (0.47%) associated with other drugs. Of the 144 cases, 74 (51.39%) received concomitant medications with pancreatotoxic potential. The likelihood of reporting of pancreatic events, compared with any other adverse event, with TCZ use was 1.32 times higher than that with other drugs. The lower bound of the 95% CI of the ROR and IC remained above the criteria of significance throughout the study period, except 2013. The findings suggest disproportionately high reporting of pancreatitis in patients receiving TCZ as compared with other drugs. This marginally high reporting is not likely to be of immediate clinical concern and needs to be interpreted cautiously.

## Introduction

Tocilizumab (TCZ) is a humanized anti-interleukin 6 (IL-6) receptor monoclonal antibody that acts by blocking IL-6/IL-6 receptor signaling^1^. IL-6 is a proinflammatory cytokine released in response to infection, trauma, and immunologic challenge; it promotes activation of T and B cells^2^. TCZ is used for treating adult patients with rheumatoid arthritis and giant cell arteritis; in children, 2 years of age or older, with polyarticular/systemic juvenile idiopathic arthritis; in adults and children for cytokine release syndrome^3^. Because of its effectiveness in treating severe life-threatening cytokine release syndrome, TCZ has been recommended as an immunotherapy for patients with extensive lung lesions or severely ill patients with elevated IL-6 levels^4^. Also, preliminary studies have reported the use of intravenous and subcutaneous TCZ in reducing the risk of mechanical ventilation or death in severe pneumonia associated with coronavirus disease 2019 (COVID-19) infection^5,6^.

Some of the common adverse effects of concern seen in clinical trials of TCZ are infections, abnormal liver function tests, neutropenia, anaphylaxis, arterial hypertension, and hypertriglyceridemia^7,8^. Few studies have also reported the occurrence of acute pancreatitis with long term use of TCZ^9–11^. However, there is a paucity of data relating the occurrence of pancreatitis following TCZ use. A recent case report highlighted acute hypertriglyceridemia with elevated inflammatory biomarkers, consistent with acute pancreatitis, in patients with COVID‐19 treated with TCZ^12^; hence, the long-term use of TCZ for systemic inflammatory disorders and acute use in a large population for COVID-19 necessitates the evaluation of the potential likelihood of pancreatitis, a condition with potentially serious health consequences, following its use.

Adverse event databases form a good data source for the initial exploration of a link between a suspected drug and the adverse event^13^. Although the results are not confirmatory, they help decide whether resource-intensive interventions need to be employed to further explore the drug-adverse event relationship. In this study, using the United States Food and Drug Administration (US FDA) Adverse Event Reporting System (FAERS) database, we intend to determine whether pancreatitis is reported more often with TCZ use compared with other drugs, by performing a disproportionality analysis and describe the clinical and demographic characteristics of the cases. Increased reporting of pancreatitis following TCZ use indicates the need for a thorough investigation of the drug-adverse event link and continued monitoring and evaluation of such cases. One of the major drawbacks of the FAERS database is the presence of duplicate case reports. An as–is use of the database without excluding duplicate reports is likely to significantly affect the study results^14^. We overcome this drawback by employing a deduplication procedure along with systematic extraction of cases with the drug-adverse event combination to enhance the data reliability.

## Methods

This was a retrospective database study; we used the United States FAERS, a database containing the adverse event reports submitted to FDA by sources such as healthcare professionals, consumers, and drug manufacturers^15^. The database has been used extensively for detecting potential drug safety issues. The study protocol was approved by Kasturba Medical College Institutional Ethics Committee (IECKMCMLR-08/2020/229), and since the FAERS is a public database containing de-identified data, the need for informed consent was waived.

We downloaded the quarterly raw data files of individual case safety reports (ICSR) in ASCII format for the period January 2013 to December 2019. We began with year 2013 since the FAERS was launched towards the end of 2012, replacing the legacy AERS system; also, the subcutaneous formulation of TCZ received approval in United States in 2013^3^. The adverse event data of an ICSR are present in a set of seven files containing demographic information of the patient (DEMO), information regarding all the drugs reported for the event (DRUG), the Medical Dictionary for Regulatory Activities (MedDRA) terms coded for the adverse reactions (REAC), the adverse event outcome (OUTC), report source (RPSR), drug therapy start and end dates (THER), and the MedDRA terms coded for the indications for drug use (INDI)^15^. The data from the downloaded dollar sign-limited text files were imported into Microsoft Excel spreadsheets for further processing.

In the FAERS, each ICSR is assigned a unique identification number (Primary ID), which is a concatenation of the case ID and the version history number. To avoid counting different versions (follow-up reports) of the same adverse event report, we performed deduplication as proposed by the FDA; this involves retaining only one version of the adverse event and excluding all other versions^15^. However, the same adverse event may be reported by different sources at different time periods, and the FDA processing logic may fail to identify the two reports as the same. For example, the same adverse event reported by both the patient and the drug manufacturer may be assigned different identification numbers, thereby erroneously increasing the number of events counted. One of the measures proposed to eliminate such duplicate cases is to look for matching information in multiple data fields in the ICSR. A proposed method for identifying duplicate cases is based on matching data in the event date, age, sex, and reporter country fields^16^. However, many case reports may have one or more of these fields empty, which increases the chances of these reports being considered a duplicate; for example, if all the data fields, except for the event date, are empty, two reports containing the same event date will be identified as duplicates. To overcome this problem, along with the abovementioned fields, we also included the suspect drug name(s) and the reaction(s) fields to match cases^16^. Further, to avoid missing detection of duplicates due to differences in the letter case, the order of drug names/reaction, presence of non-alphanumeric characters, we used a process of word concatenation, capitalization of letters, removal of non-alphabetical characters, and sorting of this concatenated data alphabetically using the functionalities in Microsoft Excel program to improve detection of duplicate data. The initial deduplication based on the case identification numbers was performed using the DEMO files. We added the data from the drug and reaction fields (extracted from DRUG and REAC files), after processing them as mentioned above, into the DEMO file to perform the second deduplication step of comparing multiple data fields. This deduplicated list of ICSRs was then used to extract the relevant data and perform the disproportionality analysis.

Following deduplication, we searched for adverse event reports of pancreatitis using the following MedDRA terminology preferred terms: pancreatic duct obstruction; obstructive pancreatitis; immune-mediated pancreatitis; pancreatic toxicity; oedematous pancreatitis; pancreatic necrosis; pancreatic duct stenosis; pancreatic infarction; ischaemic pancreatitis; pancreatitis; pancreatitis acute; pancreatitis haemorrhagic; pancreatitis necrotising; hemorrhagic necrotic pancreatitis; pancreatic failure; and pancreatic haemorrhage. We chose these terms based on a literature search to identify the various adverse event and pathological terms used in the published literature to describe the toxic effects of drugs on pancreas^17–19^.

We also searched for adverse events reported by doctors, pharmacists, or other healthcare professionals where TCZ was mentioned as the primary or secondary suspect medication for the reported event. The brand names as well as the generic name, i.e. Tocilizumab, Actemra, Actemra actpen, and Roactemra, were used to search for the relevant cases. Reports which included at least one of the preferred terms for pancreatitis and listed TCZ as a primary or secondary suspect medication were assumed to be cases of TCZ-induced pancreatitis. We also recorded the number of adverse events other than pancreatitis that were reported with TCZ. Similarly, we determined the number of cases of pancreatitis reported in association with all other drugs in the FAERS database, other than TCZ, and the total number of other adverse events reported during the study period. Since many other drugs have pancreatotoxic potential, we noted the concomitant medications received by the cases; we determined the number of cases with use of such pancreatotoxic concomitant medications. The classification proposed by Badalov et al. was used to identify the pancreatotoxic drugs^20^.

### Disproportionality analysis

A disproportionality analysis determines whether there is disproportional reporting of a drug-adverse event combination compared with the occurrence of the adverse event with other drugs in the database^21^. Our study objective was to determine whether the reporting of pancreatitis was disproportionately high in association with TCZ use. The disproportionality methods used in our study are the reporting odds ratio (ROR) and information component (IC) with shrinkage, which are commonly used data mining methods for signal detection^22–24^. The calculation of disproportionality measures is based on a two-by-two contingency table (Supplementary Table S1)^21^. ROR is the ratio of the odds of an adverse event in those who did by those who did not receive the suspected medication. IC involves the calculation of the observed to expected ratio; since it may provide erroneous results when the observed or expected number is extremely low, a shrinkage factor is applied to the numerator and denominator which moderates the measure towards a null value in the absence of a true difference (Supplementary Table S1). A ROR with the lower bound 95% confidence interval (CI) > 1 and a lower limit of a two-sided 95% interval of IC (IC_025_) more than zero was considered significant, indicating that the number of events (pancreatitis) observed with the suspect drug (tocilizumab) is more than the number expected by chance alone^22,24^. Disproportionality analysis was conducted for pancreatitis inclusive of all the preferred terms and also for each preferred term when the number of cases was ≥ 5.

### Statistical analysis

The clinical and demographic characteristics of the cases with suspected TCZ-induced pancreatitis have been reported as median (interquartile range [IQR]) since the data were not normally distributed (Shapiro–Wilk test, p < 0.05). Categorical variables are reported as proportion and percentage. The descriptive statistics were calculated using Statistical Package for Social Sciences, version 11.5 (SPSS Inc., Chicago, USA).

## Results

During the period 2013–2019, 9,122,256 adverse event reports were received by FAERS. Following case deduplication, the total number of reports was 3,383,910. Among these, 144 (0.004%) case reports were of pancreatic adverse events associated with TCZ use, and 15,907 (0.47%) associated with use of other drugs. Apart from the pancreatic adverse events, 22,899 other adverse events were reported with TCZ as a primary or secondary suspect medication.

The demographic and clinical characteristics of the 144 cases of pancreatic adverse events associated with TCZ use are shown in Table 1. The median age of the patients was 56 years (IQR, 45.75–66.50); females represented 53% of the cases. The most common reported pancreatic adverse event was pancreatitis. Majority of the cases fulfilled the criteria for a serious adverse event. The median duration of onset from the time of initiation of TCZ was 4 months (IQR, 1–10.75). Concomitant medications were reported in 107 cases, and among these, 74 (51.39%) patients received drugs that can cause pancreatitis; 38 patients received more than one such drug, besides TCZ (Table 2). Among the 21 countries which reported TCZ-associated pancreatic events to FAERS, the highest number was reported from the United States (19%).

**Table 1:** Characteristics of cases with pancreatic adverse events following tocilizumab use.

Characteristics	Value
**Age, median (IQR)**	
All	56 (45.75–66.50)
Male	56 (43–70)
Female	56.5 (48.50–66.50)
**Gender, N (%)**	
Male	47 (32.64)
Female	76 (52.78)
Not specified	21 (14.58)
**Adverse event outcome, N (%)**	
Died	14 (9.72)
Hospitalized	85 (59.03)
Life threatening	2 (1.39)
Other outcomes	43 (29.86)
**Adverse event terms, N (%)**^**a**^	
Pancreatitis	90 (62.50)
Pancreatitis acute	35 (24.31)
Pancreatic toxicity	8 (5.56)
Pancreatic failure	6 (4.17)
Pancreatic necrotising	5 (3.47)
Pancreatic necrosis	1 (0.69
Pancreatitis haemorrhagic	1 (0.69)
Pancreatolithiasis	1 (0.69)
**Reporting country, N (%)**	
United States	27 (18.75)
Canada	23 (15.97)
Germany	25 (17.36)
France	19 (13.19)
Japan	10 (6.94)
United Kingdom	8 (5.56)
Others	32 (22.22)
**Medications, N (%)**	
Tocilizumab only	37 (25.69)
Tocilizumab + other drugs	107 (74.31)
Tocilizumab + other pancreatotoxic drugs	74 (51.39)

^a^Total count exceeds 144 since three reports contained two adverse event terms each.

**Table 2:** Concomitant drugs with potential to cause pancreatitis received by patients with tocilizumab-associated pancreatic adverse events^20^.

Class Ia	Class Ib	Class II	Class III	Class IV
Codeine	Azathioprine	Acetaminophen	Alendronate	Cyclophosphamide
Enalapril	Dexamethasone	Asparaginase	Atorvastatin	Diclofenac
Furosemide	Ethinyl estradiol	Propofol	Ceftriaxone	Ketoprofen
Mesalamine	Losartan		Cyclosporine	Lovastatin
Metronidazole	Mercaptopurine		Hydrochlorothiazide	Ramipril
Simvastatin	Omeprazole		Indomethacin	Ritonavir
Valproic acid	Conjugated estrogens		Lisinopril	Rosuvastatin
	Cotrimoxazole		Metformin	Tacrolimus
			Naproxen	
			Prednisone	
			Prednisolone	

The drugs have been listed in a particular class based on the classification of drugs causing acute pancreatitis suggested by Badalov et al.^20^, which is based on the level of evidence available.

The disproportionality analysis results are shown in Table 3. The ROR and IC for the entire study data, considering all the reported pancreatic adverse event terms together, fulfilled the significance criteria for disproportionality. The likelihood of reporting of pancreatic events, compared with any other adverse event, with TCZ use was 1.32 times higher than that with other drugs; the Bayesian IC also indicates that the reporting of the drug-adverse event combination of interest is slightly higher than that expected when there is no association between the drug and event. On analyzing individual pancreatic adverse event terms, the disproportionality statistic, both ROR and IC, for the terms pancreatic toxicity, pancreatitis, and pancreatic failure were significant. While there were 90 cases of pancreatitis reported during the study period, there were only 8 and 6 cases of pancreatic toxicity and pancreatic failure, respectively, associated with TCZ use. The highest number of cases were reported to FAERS in 2018; the disproportionality analysis for each year is shown in Table 4. The IC_025_ was below the criteria of significance for all the years, except 2016. In contrast, the lower bound of the 95% confidence interval of ROR was above the criteria of significance for all the years, except 2017 and 2019. However, the magnitude of increased reporting was less than twice the number that would be expected in the absence of any association between the drug and the adverse event.

**Table 3:** Disproportionality analysis for pancreatitis associated with tocilizumab use.

MedDRA term (notation)	ROR	95% CI	IC	95% CI
Pancreatitis [all terms]	1.32	1.12–1.56^a^	0.39	0.12–0.59^a^
Pancreatitis (10033645)	1.38	1.12–1.70^a^	0.45	0.11–0.71^a^
Pancreatitis acute (10033647)	0.93	0.66–1.29	− 0.11	− 0.67 to 0.29
Pancreatic toxicity (10076205)	53.06	23.62–119.18^a^	3.59	2.38–4.40^a^
Pancreatic failure (10079281)	5.72	2.53–12.94^a^	2.04	0.62–2.95^a^
Pancreatitis necrotising (10033654)	1.78	0.74–4.31	0.73	− 0.83 to 1.71

*CI* confidence interval, *IC* information component, *MedDRA* medical dictionary for regulatory activities, *ROR* reporting odds ratio.

^a^Statistically significant.

**Table 4:** Year-wise disproportionality analysis for pancreatitis associated with tocilizumab use.

Year	Number of cases	ROR	95% CI	IC	95% CI
2013	19	1.64	1.04–2.59^a^	0.68	− 0.09 to 1.24
2014	16	1.70	1.04–2.79^a^	0.73	− 0.12 to 1.33
2015	18	1.76	1.10–2.80^a^	0.77	− 0.02 to 1.35
2016	23	1.83	1.21–2.77^a^	0.84	0.14–1.35^a^
2017	21	1.28	0.83–1.96	0.34	− 0.39 to 0.87
2018	31	1.49	1.04–2.12^a^	0.55	− 0.05 to 0.99
2019	16	0.64	0.39–1.04	− 0.63	− 1.47 to − 0.02

*CI* confidence interval, *IC* information component, *ROR* reporting odds ratio.

^a^Statistically significant.

**Figure 1:**
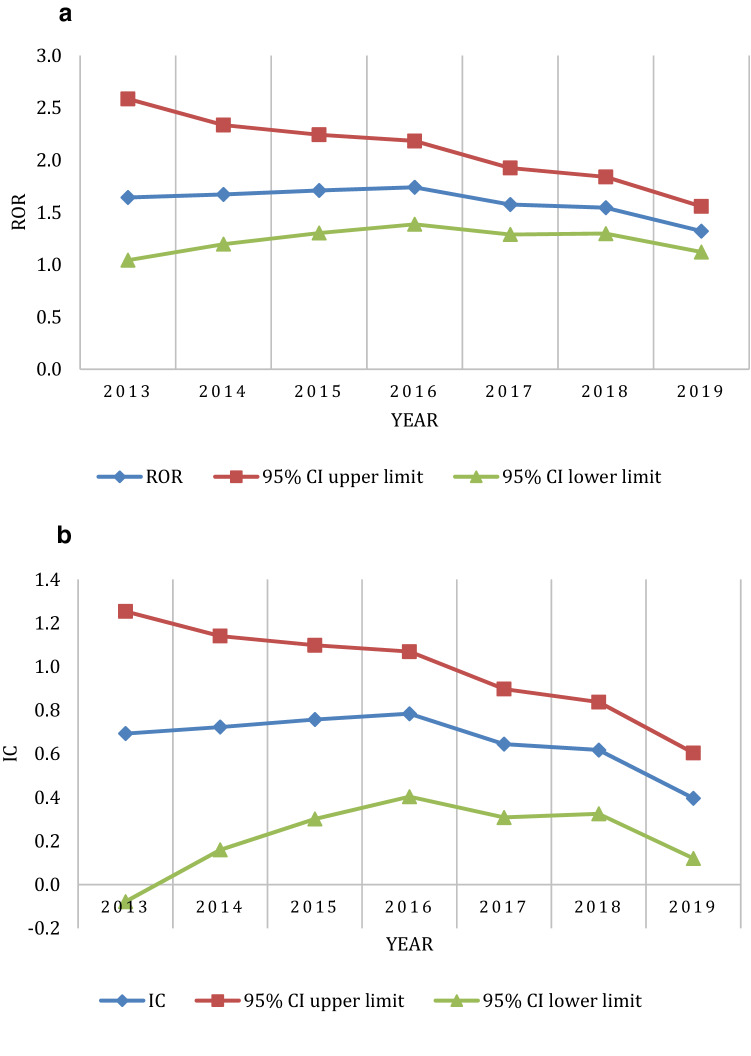
Cumulative change in the reporting odds ratio (ROR) (**a**) and information component (IC) (**b**) values for suspected tocilizumab-induced pancreatitis reported to United States food and drug administration adverse event database from 2013 to 2019.

To determine the change in the disproportionality statistics over time, we calculated the cumulative ROR and IC over time, from 2013 to 2019 (Fig. 1). The lower bound of the 95% confidence interval of the ROR remained above 1 throughout the study period, with a progressive narrowing of the confidence intervals. A similar trend is seen with regard to IC_025_ with the exception that the lower bound of the 2013 IC was below the criteria of significance. To determine the disproportionality results in the absence of deduplication, we performed an unplanned analysis of the FAERS raw data by running multiple drug and adverse event search term queries (considering all pancreatic adverse event terms together) in the FAERS public dashboard^25^; the resultant ROR was 2.07 (1.82–2.35) and IC 1.03 (0.82–1.19). We then used AERSMine data mining software program to determine if the concomitant administration of pancreatotoxic drugs, other than TCZ, had an influence on the disproportionality results^26^. AERSMine program performs ontological aggregation of the various drug names, disease conditions, and adverse event terms^26^. The program also allows dividing the cases into cohorts based on drugs received/to be excluded, adverse events of interest, etc. We divided the cases in to those who received TCZ but not any of the other potentially pancreatotoxic drugs and those who received TCZ with at least one of the pancreatotoxic drugs. The resultant ROR and IC for those who did versus those who did not receive concomitant pancreatotoxic drug(s) were 0.95 (0.79–1.14) and − 0.07 (− 0.37 to 0.14) versus 1.48 (1.19–1.83) and 0.55 (0.19–0.81), respectively.

## Discussion

Our analysis of the FAERS database for the period 2013–2019 showed that the reporting of pancreatitis in association with TCZ use was marginally higher, less than double the number, compared with that in association with other drugs in the database. The disproportionality analysis shows that the marginally higher reporting has remained at almost the same level during the 7-year period studied, with a gradual narrowing of the confidence intervals. Our study data shows that the disproportionality statistic was higher in the pre-2017 period^27^, albeit with a wider confidence interval. Considering the large number of patients exposed to the drug every year, this small but significant disproportionality finding is important, but at the same time, this needs to be considered taking into account the inherent limitations of data obtained from adverse event databases such as FAERS used in the current study^15,28^. It is also to be noted that the results did not show increased reporting of acute pancreatitis, which is more likely to be drug-related, with TCZ.

Pancreatitis is a serious condition with significant potential morbidity and mortality. Drugs are a relatively rare cause of acute pancreatitis, responsible for 3–5% of the cases^17,29–31^. Most drug-induced pancreatitis cases are mild to moderate in severity; however, failure to implicate a drug as a causative agent can result in serious complications^27^. A 52-week clinical, functional, and radiographic efficacy and safety study of TCZ in patients with rheumatoid arthritis as well as a few case reports have reported the occurrence of pancreatitis^9–11,32^. In contrast, retrospective cohort studies have shown that rheumatoid arthritis by itself can increase the risk of acute pancreatitis, irrespective of the use of disease modifying antirheumatic drugs, which makes ascertaining the cause more difficult^33,34^. Other autoimmune diseases, such as systemic lupus erythematosus, have also been shown to cause pancreatitis, although uncommon, and the reported estimates are likely to be highly variable^35^. The mechanism by which drugs, in general, cause pancreatitis is not clearly understood. A number of mechanisms have been postulated, such as direct cytotoxic tissue injury^36^; indirect metabolic effects such as hypercalcemia and hypertriglyceridemia^37,38^; idiosyncratic or hypersensitivity reactions^39^; localized angioedema^40^; arteriolar thrombosis^37^. The exact mechanism for acute pancreatitis with TCZ is unknown, but it may be due to hypertriglyceridemia and IL-6 inhibition^11^. The likely duration of treatment with TCZ for onset of pancreatitis is unclear. In a study by Flaig et al.^11^, pancreatitis occurred after the administration of second dose, where the first dose was given 2 weeks before the second dose. In a case report by Alfreijat et al.^32^, TCZ was given 3 months before the onset of hepatitis and mild pancreatitis at a dose of 8 mg/kg/month, with the last injection 1 week before the hospitalization. One more case report described increased serum triglyceride levels and acute pancreatitis biomarkers on the third day of treatment with TCZ used to treat COVID-19 infection^12^. Hence, with the available studies, it is difficult to rule out the possibility of acute pancreatitis when TCZ is used for a short duration as for treating COVID-19 infection. In the current study, the median duration of onset was 4 months; however, data was missing or incomplete for a large number of cases.

Our study results show that TCZ-associated pancreatitis was reported more in females, although the validity of this finding cannot be confirmed, considering the wide range of factors that influence adverse event spontaneous reporting. There is some evidence, however, that females may be affected more often^29,41^. Majority of the reported cases were serious adverse events, with approximately 10% mortality. However, many of these patients had comorbid events which could have contributed to the case severity and outcome. In addition, approximately 51% of the cases received concomitant medications with a pancreatotoxic potential. Although the presence of concomitant medication with pancreatotoxic potential makes it difficult to attribute the adverse events to TCZ alone, our analysis using AERSMine^26^ showed that disproportionate reporting was seen only in the cohort who received TCZ without concomitant pancreatotoxic drugs. Badalov et al. proposed a 4-class classification of drugs implicated in causing pancreatitis^20^. Accordingly, TCZ may be classified as a class II drug, in which 75% of at least four reported case reports describe a latency period and no rechallenge has been done^42^; 16 reported concomitant medications belonged to class I.

We used the ROR, a frequentist approach, and IC, a Bayesian approach, to determine the statistical significance of the obtained results. While each approach has specific advantages and disadvantages, both the statistic are expected to provide congruent results^43^. This is true in our study where both the disproportionality measures show a small, but significant, disproportionately higher reporting of pancreatitis in patients who received TCZ. While the cumulative change in the disproportionality measures in our study show the gradual decrease in the confidence interval with a significant statistic, the yearly analysis shows that both measures fulfilled the criteria of significance only in the year 2016. This may be due to the small overall number of cases reported each year, resulting in a wide confidence interval, which failed to meet the significance criteria. The fact that the significance criteria was met for only one year as per IC and four of the studied years as per ROR highlights the possible differences in the interpretation of the data in situations where the number of events reported is low relative to the database size based on the disproportionality measure adopted. ROR has been adopted as the disproportionality statistic by the EudraVigilance system based on the findings of the PROTECT project^22^, while IC is the disproportionality statistic used by World Health Organization-Uppsala Monitoring Centre^23^. ROR has the advantage of simplicity and easier understanding of the output whereas IC avoids overestimation of results/spurious results in situations where the expected and observed counts are small^44^. Despite the differences, all disproportionality statistic are based on a two-by-two contingency table, and largely provide similar results, with the choice of a particular statistic being dictated by the availability of resources, implementation, and the choice of communication of results^43^.

The presence of duplicate case reports is common and can significantly affect the disproportionality analysis outcomes^45^. We used the routine deduplication procedure based on case IDs supplemented with checking of matching data in multiple fields of the case report; however, this procedure does not identify duplicate reports that do not contain matching data due to spelling errors or exclusion/inclusion of drug names/event terms by two reporters reporting the same event. In fact, we did find potentially duplicate cases among the 144 cases obtained after deduplication; for example, multiple potential duplicate cases of a pancreatic failure event were identified, each case differing due to mismatch in one data field. While we manually processed each data file using the functionalities in MS Excel, other more automated but technically complex procedures have been described and implemented^16^, including an algorithm that can potentially overcome the aforementioned limitations^46^. To identify the magnitude by which our results differ from the analysis of the unprocessed data from the raw files, we performed an analysis using the FAERS public dashboard; as expected, both the ROR and IC were much higher than that obtained following deduplication. We extracted the cases reporting the drug-adverse event pair and subjected it to deduplication procedure adopted in the current study. This resulted in the final number of cases (i.e. 144) same as that obtained from deduplication of data from the raw files, confirming the accuracy of the procedure. There are inherent limitations of FAERS such as potential underreporting, the influence of publicity regarding an event, possible reporting errors, incomplete information, etc.^15^. Hence, the findings cannot be considered confirmatory.

To conclude, our study findings show that there is disproportionately high reporting of pancreatitis, in comparison to other adverse events, in patients receiving TCZ as compared with other drugs. However, the increased reporting is only marginally high, less than twice the number that would be expected if there is no association between the drug and the adverse event. Given the limitations of the study and the FAERS data, this marginally high reporting is not likely to be of immediate clinical concern and needs to be interpreted cautiously. Monitoring the future trends of disproportionality and published reports of pancreatitis following TCZ use are required to confirm the findings.

## Supplementary Information

Supplementary Table S1.

## Data Availability

All data pertaining to this study is available in the US FDA Adverse Event Reporting System, a public database.

## References

[1] Koike R (2009). Japan College of Rheumatology 2009 guidelines for the use of tocilizumab, a humanized anti-interleukin-6 receptor monoclonal antibody, in rheumatoid arthritis. Mod. Rheumatol..

[2] Sebba A (2008). Tocilizumab the first interleukin-6-receptor inhibitor. Am. J. Health Syst. Pharm..

[3] Actemra. United States Food and Drug Administration. https://www.accessdata.fda.gov/drugsatfda_docs/label/2019/125276s127,125472s040lbl.pdf (Accessed 26 September 2020).

[4] Chen ZR (2020). Pharmacotherapics advice in guidelines for COVID-19. Front. Pharmacol..

[5] Guaraldi G (2020). Tocilizumab in patients with severe COVID-19: A retrospective cohort study. Lancet Rheumatol..

[6] Kewana T (2020). Tocilizumab for treatment of patients with severe COVID-19: A retrospective cohort study. EClinicalMedicine..

[7] Jones G, Ding C (2010). Tocilizumab: A review of its safety and efficacy in rheumatoid arthritis. Clin. Med. Insights Arthritis Musculoskelet. Disord..

[8] Giles JT (2020). Cardiovascular safety of tocilizumab versus etanercept in rheumatoid arthritis: A randomized controlled trial. Arthritis Rheumatol..

[9] Takeuchi T (2011). Clinical, radiographic and functional effectiveness of tocilizumab for rheumatoid arthritis patients—REACTION 52-week study. Rheumatology.

[10] Parekh PJ, Howerton D, Johnson DA (2013). Not your everyday case of acute pancreatitis: A rare complication of a common diagnosis. ACG Case Rep. J..

[11] Flaig T (2016). Tocilizumab-induced pancreatitis: Case report and review of data from the FDA Adverse Event Reporting System. J. Clin. Pharm. Ther..

[12] Morrison AR (2020). Acute hypertriglyceridemia in patients with COVID-19 receiving tocilizumab. J. Med. Virol.

[13] Sharrar RG, Dieck GS (2013). Monitoring product safety in the postmarketing environment. Ther. Adv. Drug Saf..

[14] Norén GN (2017). The power of the case narrative—Can it be brought to bear on duplicate detection?. Drug Saf..

[15] Questions and Answers on FDA's Adverse Event Reporting System (FAERS). U.S. Food and Drug Administration. https://www.fda.gov/drugs/surveillance/questions-and-answers-fdas-adverse-event-reporting-system-faers (Accessed 26 April 2021).

[16] Banda JM (2016). A curated and standardized adverse drug event resource to accelerate drug safety research. Sci. Data..

[17] Jones MR, Hall OM, Kaye AM, Kaye AD (2015). Drug-induced acute pancreatitis: A review. Ochsner J..

[18] Hung WY, AbreuLanfranco O (2014). Contemporary review of drug-induced pancreatitis: A different perspective. World J. Gastrointest. Pathophysiol..

[19] Vege, S. S. Etiology of acute pancreatitis. In: T. W. Post, Whitcomb, D. C., Grover, S. (Eds), UpToDate. https://www.uptodate.com/contents/etiology-of-acute-pancreatitis (Accessed 26 April 2021).

[20] Badalov N (2007). Drug-induced acute pancreatitis: An evidence-based review. Clin. Gastroenterol. Hepatol..

[21] Montastruc JL, Sommet A, Bagheri H, Lapeyre-Mestre M (2011). Benefits and strengths of the disproportionality analysis for identification of adverse drug reactions in a pharmacovigilance database. Br. J. Clin. Pharmacol..

[22] European Medicines Agency. Screening for adverse reactions in EudraVigilance. 2016. https://www.ema.europa.eu/en/documents/other/screening-adverse-reactions-eudravigilance_en.pdf (Accessed 26 April 2021).

[23] Bate A (1998). A Bayesian neural network method for adverse drug reaction signal generation. Eur. J. Clin. Pharmacol..

[24] Norén GN, Hopstadius J, Bate A (2013). Shrinkage observed-to-expected ratios for robust and transparent large-scale pattern discovery. Stat. Methods Med. Res..

[25] Food and Drug Administration. FDA AEs reporting system (FAERS) public dashboard. https://www.fda.gov/Drugs/GuidanceComplianceRegulatoryInformation/Surveillance/AdverseDrugEffects/ucm070093.htm. (Accessed 30 March 2021).

[26] Sarangdhar M (2016). Data mining differential clinical outcomes associated with drug regimens using adverse event reporting data. Nat. Biotechnol..

[27] July–September 2017. Potential Signals of Serious Risks/New Safety Information Identified from the FDA Adverse Event Reporting System (FAERS). United States Food and Drug Administration. https://www.fda.gov/drugs/questions-and-answers-fdas-adverse-event-reporting-system-faers/july-september-2017-potential-signals-serious-risksnew-safety-information-identified-fda-adverse (Accessed 26 March 2021).

[28] Gould AL, Lystig TC, Lu Y, Fu H, Ma H (2015). Methods and issues to consider for detection of safety signals from spontaneous reporting databases: A report of the DIA Bayesian Safety Signal Detection Working Group. Ther. Innov. Regul. Sci..

[29] Balani AR, Grendell JH (2008). Drug-induced pancreatitis; incidence management and prevention. Drug Saf..

[30] Vinklerová I, Procházka M, Procházka V, Urbánek K (2010). Incidence, severity, and etiology of drug-induced acute pancreatitis. Dig. Dis. Sci..

[31] Weissman S (2020). Ever-increasing diversity of drug-induced pancreatitis. World J. Gastroenterol..

[32] Alfreijat M, Habibi M, Bhatia P, Bhatia A (2013). Severe hepatitis associated with tocilizumab in a patient with rheumatoid arthritis. Rheumatology (Oxford).

[33] Chang CC (2015). Increased risk of acute pancreatitis in patients with rheumatoid arthritis: A population-based cohort study. PLoS ONE.

[34] Alkhayyat M (2021). Pancreatic manifestations in rheumatoid arthritis: A national population-based study. Rheumatology (Oxford).

[35] Jadhav PP (2019). Acute pancreatitis in rheumatology practice, with emphasis on systemic lupus erythematosus: A case series and newer concepts. Indian J. Rheumatol..

[36] Thisted H (2006). Statins and the risk of acute pancreatitis: A population-based case–control study. Aliment. Pharmacol. Ther..

[37] Rünzi M, Layer P (1996). Drug-associated pancreatitis: Facts and fiction. Pancreas.

[38] Guo JJ, Jang R, Louder A, Cluxton RJ (2005). Acute pancreatitis associated with different combination therapies in patients infected with human immunodeficiency virus. Pharmacotherapy.

[39] Sinclair DB, Berg M, Breault R (2004). Valproic acid-induced pancreatitis in childhood epilepsy: Case series and review. J. Child Neurol..

[40] Eland IA (2006). Antihypertensive medication and the risk of acute pancreatitis: The European case–control study on drug-induced acute pancreatitis (EDIP). Scand. J. Gastroenterol..

[41] Barreto SG, Tiong L, Williams R (2011). Drug-induced acute pancreatitis in a cohort of 328 patients. A single-centre experience from Australia. JOP..

[42] Tenner S (2014). Drug induced acute pancreatitis: Does it exist?. World J. Gastroenterol..

[43] Wisniewski AF (2016). Good signal detection practices: Evidence from IMI PROTECT. Drug Saf..

[44] Bate A, Evans SJ (2009). Quantitative signal detection using spontaneous ADR reporting. Pharmacoepidemiol. Drug. Saf..

[45] Hauben M, Reich L, DeMicco J, Kim K (2007). 'Extreme duplication' in the US FDA adverse events reporting system database. Drug Saf..

[46] Kreimeyer K (2017). Using probabilistic record linkage of structured and unstructured data to identify duplicate cases in spontaneous adverse event reporting systems. Drug Saf..

