# Supplementary material for: Assessment of pancreatitis associated with tocilizumab use using the United States Food and Drug Administration Adverse Event Reporting System database
Source: Sci Rep. 2021 Sep 22;11:18818. doi: 10.1038/s41598-021-98325-w (PMC8458491; doi:10.1038/s41598-021-98325-w)
Supplement: Supplementary file 1 — Supplementary Table S1. [file 41598_2021_98325_MOESM1_ESM.docx]

**Supplementary Table S1.** Determining the disproportionality measures using a two-by-two contingency table

|  | **Developed pancreatitis** | **Developed adverse event(s) other than pancreatitis** |  |
| --- | --- | --- | --- |
| **Received Tocilizumab** | a | b | a + b = P |
| **Received drug(s) other than Tocilizumab** | c | d | c+ d = Q |
|  | a+ c = R | b+ d = S | a + b + c + d = T |

Reporting odds ratio = [a/b] / [c/d]

Observed value (O) = a

Expected value (E) = [R x P] / T

Information component = log_2_ ([O + ½] / [E + ½])
